# Maxillary Immediate Molar Implant Placement Into Palatal Root Socket: A Case Report of Two-Year Follow-Up

**DOI:** 10.7759/cureus.71152

**Published:** 2024-10-09

**Authors:** Guanqi Liu, Runheng Liu

**Affiliations:** 1 Dentistry, Sun Yat-sen University, Guangzhou, CHN; 2 Dentistry, Hospital of Stomatology, Guanghua School of Stomatology, Sun Yat-sen University, Guangzhou, CHN

**Keywords:** immediate implant, maxillary molar, minimally invasive technique, palatal root socket, primary stability

## Abstract

The palatal root of the maxillary molar is the largest among the three roots; therefore, the direction of the palatal root extraction socket can serve as a choice for the implantation direction during immediate implant placement. By directly implanting the implant into the extraction socket of the palatal root of the maxillary molar, the procedure of immediate implantation of maxillary molars can be simplified, the treatment period can be shortened, costs can be reduced, surgical trauma can be minimized, and some significant risks and complications associated with sinus augmentation surgery can be avoided. This case report, with a two-year follow-up, details the process of immediate implant placement into the palatal root socket of the maxillary molar.

## Introduction

Since Professor Willi Schulte first proposed the concept of immediate implantation in 1978, a wealth of literature has confirmed its advantages: fewer surgical interventions and a shorter treatment duration [1]. However, immediate implantation in the molar area presents greater surgical challenges, particularly in achieving primary stability and effective wound closure [2,3]. This case report details a case of immediate implantation in the maxillary molar region, where the implant was strategically placed into the palatal root extraction socket to ensure adequate primary stability. Furthermore, by tailoring the implant carrier to fabricate a personalized healing abutment, the wound was successfully closed. A 2-year follow-up showed favorable clinical results.

## Case presentation

**Figure 1:**
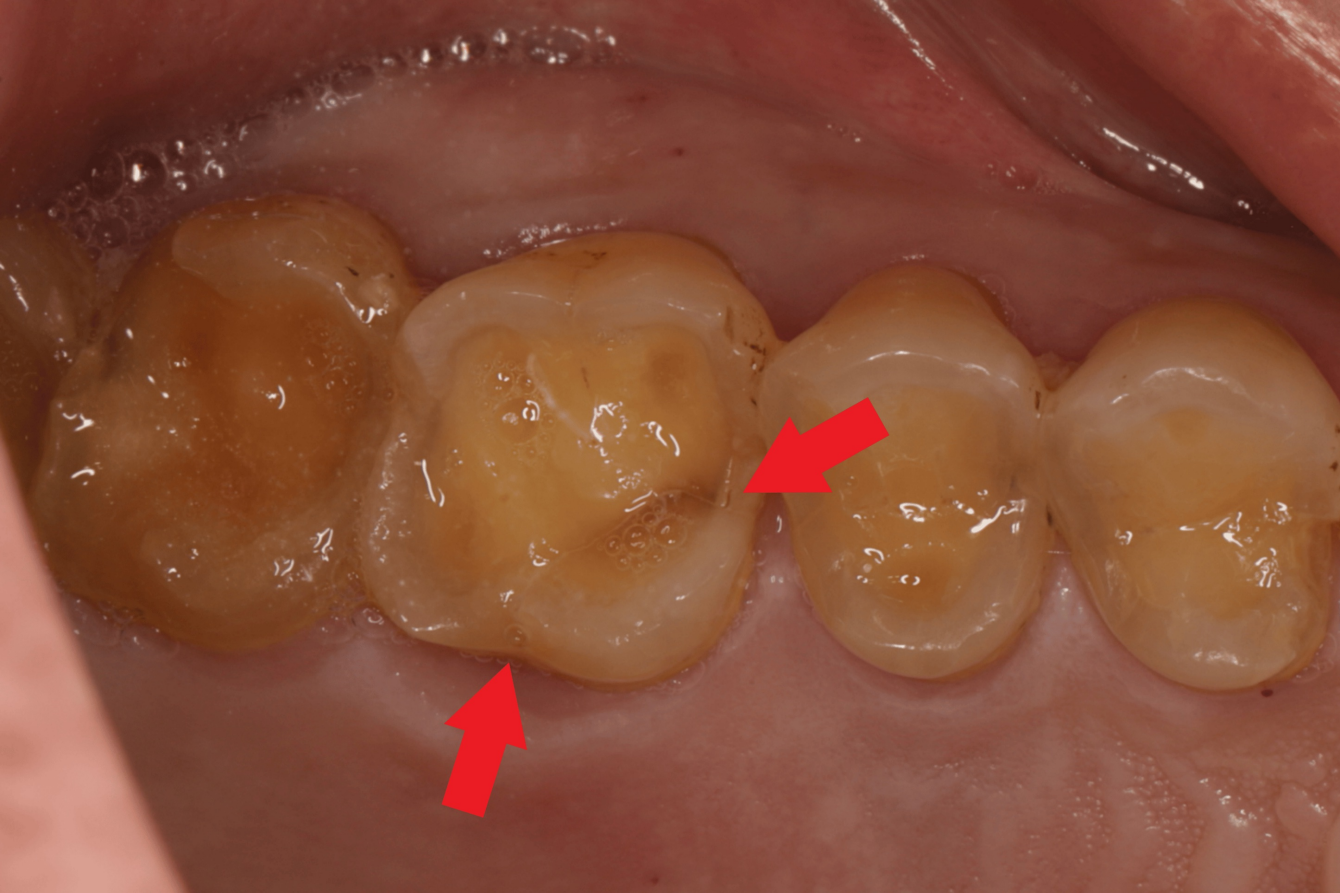
Cracks visible upon intraoral examination (indicated by the red arrow).

**Figure 2:**
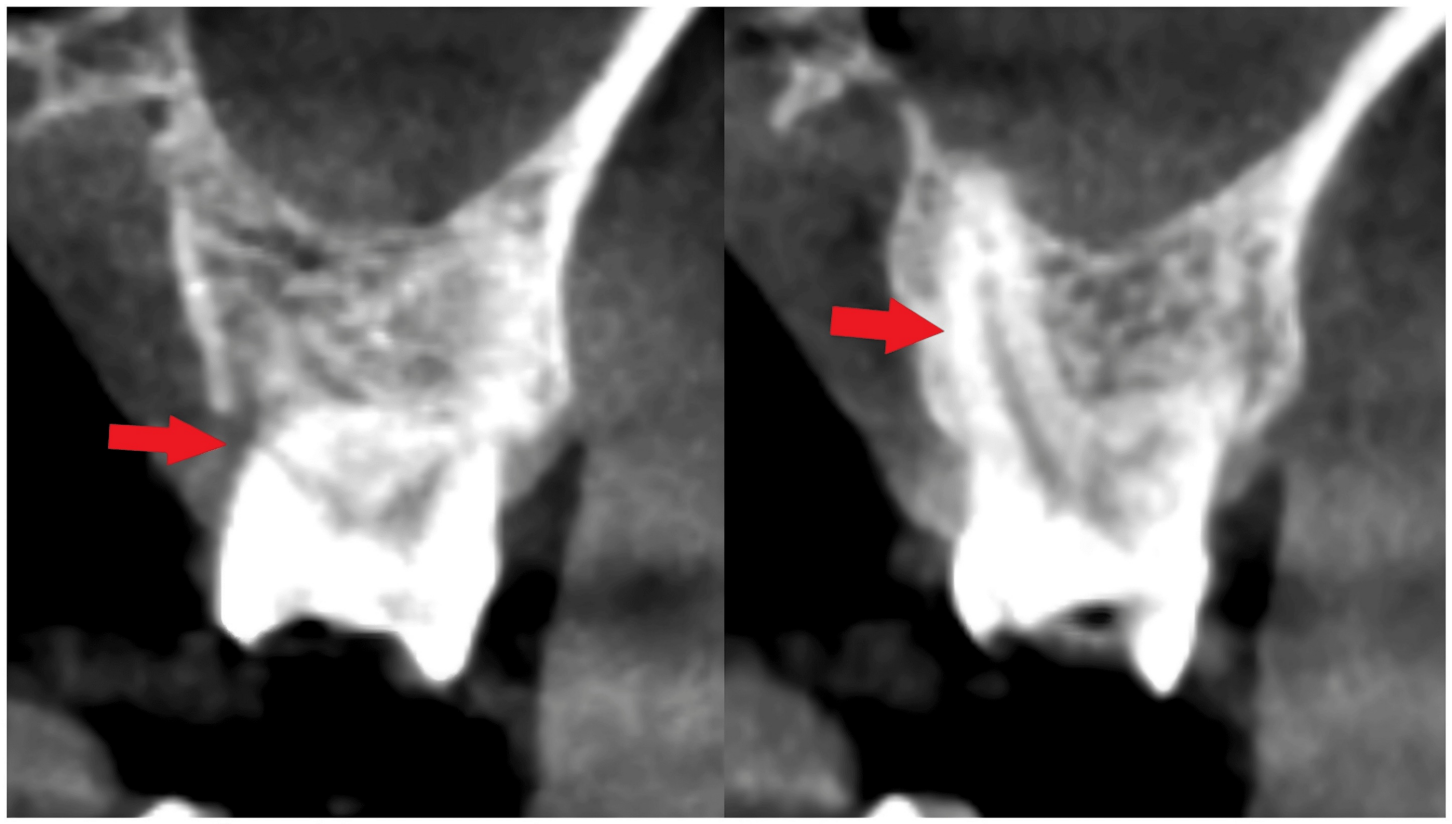
Preoperative CBCT examination. (A) Visible crack (indicated by red arrow); (B) Palatal root of maxillary molar.

A 63-year-old man came with a chief complaint of pain at tooth no. 26 due to a crown fracture. Clinical examination revealed tooth no. 26 shows severe wear on the occlusal surface, with cracks visible in the mesial groove and distal lingual groove (Figure 1). A cone beam computed tomography (CBCT) scan was performed, which confirmed a crown fracture of tooth no. 26, with the crack depth being level with the palatal bone. The length of the palatal root is approximately 10 mm, the diameter in the middle of the palatal root is about 3.7 mm, and the diameter at the apex of the palatal root is about 2.5 mm (Figure 2).

Treatment options discussed included delayed implant placement and immediate implant placement. The patient wished to have the tooth extracted, and the implant surgery performed as soon as possible and requested, minimally invasive treatment. Therefore, it was decided with the patient's consent to proceed with immediate maxillary posterior teeth implant placement.

**Figure 3:**
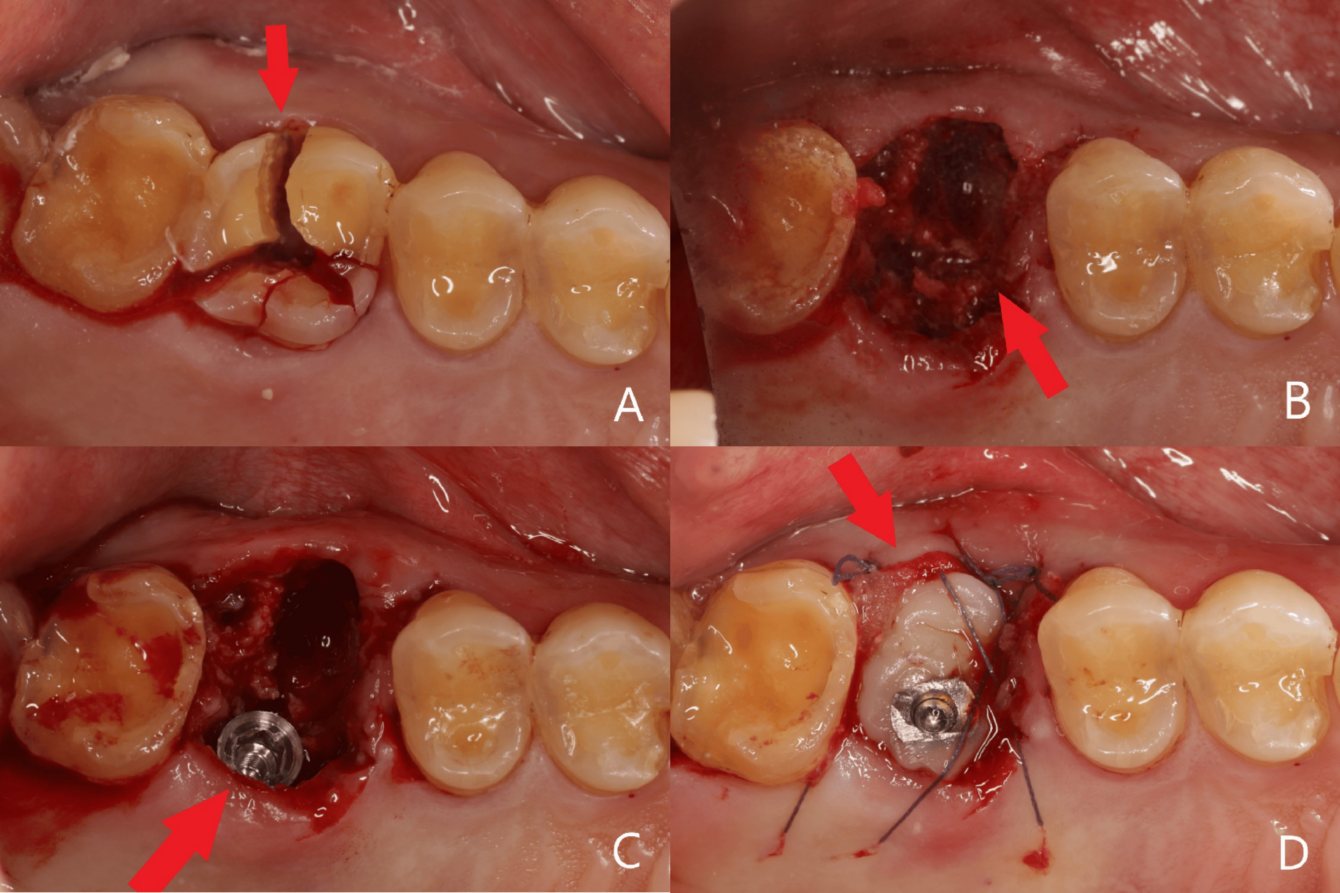
Surgical procedure (A) Tooth no. 26 is trisected into its individual roots; (B) partial septal bone is damaged; (C) implant is placed into palatal root socket; (D) customized healing abutment for socket sealing and wound closure.

Due to root ankylosis, the tooth extraction was not straightforward, resulting in partial damage to the septum bone. However, based on the measurement data from the palatal root, it is feasible to place the implant directly into the palatal root socket. Utilizing a maxillary sinus floor elevation instrument through the palatal root socket to perforate the maxillary sinus floor. A Zimmer TSV dental implant (4.1 mm×10 mm) was placed into the palatal root socket with an implant insertion torque exceeding 35 N·cm. Bio-Oss collagen was filled into the extraction sockets of both the mesial and distal buccal roots. The implant carrier was used to fabricate a custom healing abutment and close the wound (Figure 3).

**Figure 4:**
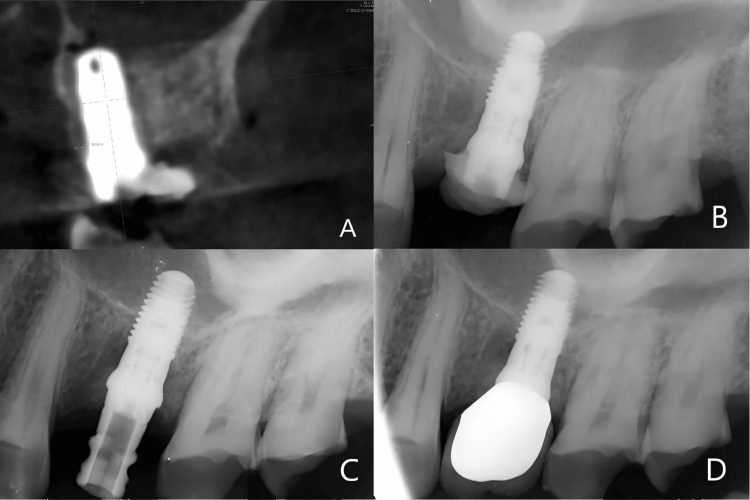
RadiRadiologic examination A: Cross-sectional view of postoperative CBCT; B: Periapical radiography one month after surgery; C: Periapical radiography 3.5 month after surgery; D: Periapical radiography 4.5 month after surgery.

Postoperative CBCT showed that the implant was placed into the palatal root socket of tooth no. 26 with the apical end penetrating into the maxillary sinus (Figure 4A). At the 1-month postoperative follow-up, periapical radiographs indicated no abnormalities (Figure 4B). At 3.5 months post-surgery, an impression was taken for the implant (Figure 4C). At 4.5 months post-surgery, the implant crown was placed, and periapical radiographs indicated good osseointegration of the implant, with the crown in place and no other abnormalities (Figure 4D).

**Figure 5:**
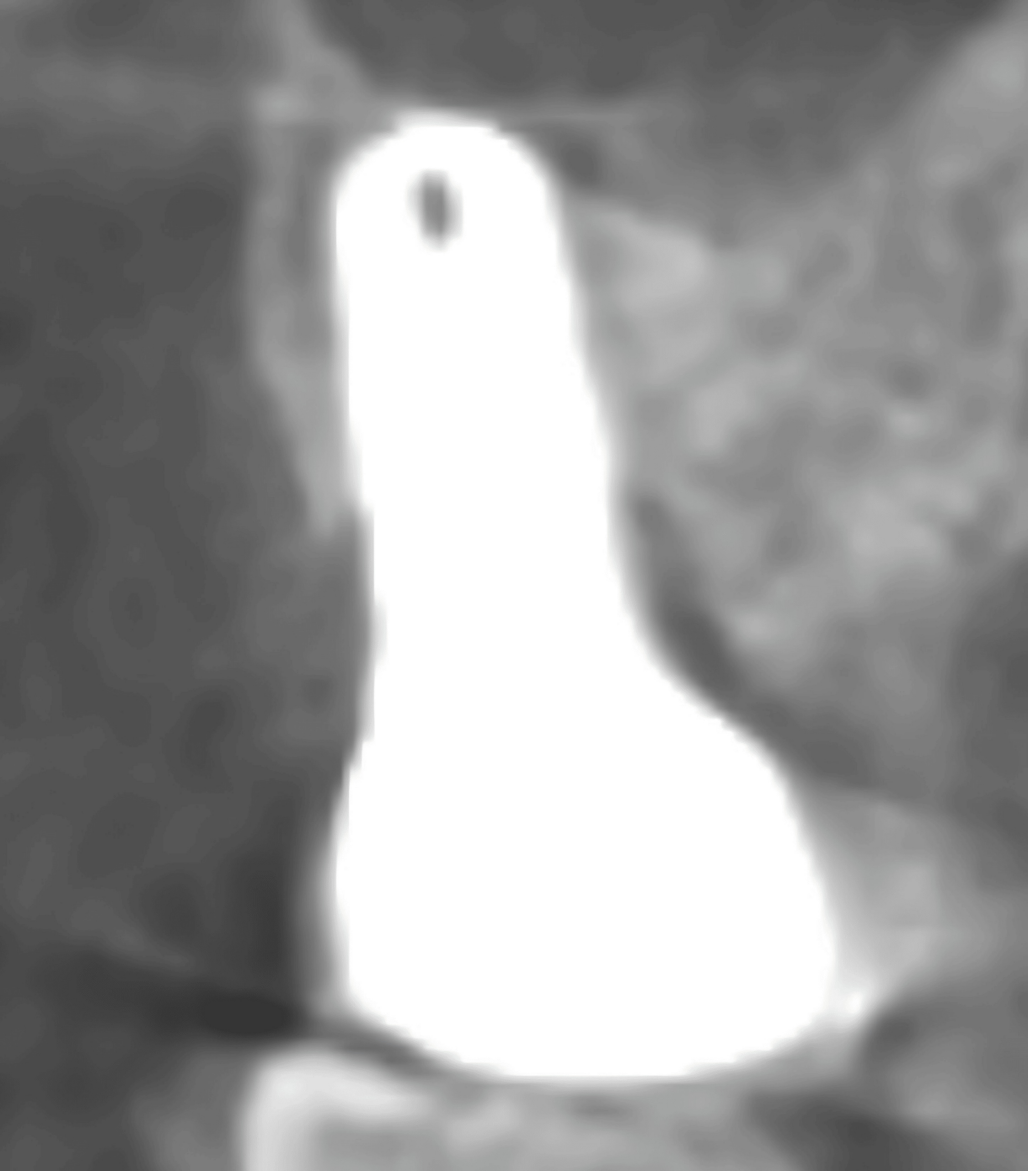
Cross-sectional view of two-year follow-up CBCT

A two-year follow-up CBCT showed that the palatal bone thickness of the implant was >1mm (Figure 5). 

## Discussion

Several critical clinical determinants influence the success of immediate molar implantation (IMI). Notably, the morphology of the alveolar socket is a pivotal factor that can ascertain the stability of an IMI [2,4]. The ideal alignment for immediate molar implant placement, tailored to prosthetic requirements, is typically accomplished when the implant osteotomy is meticulously crafted within the inter-radicular/furcation bone region (IRS) [1,5]. Nonetheless, when the clinician determines that the volume of inter-radicular/furcation bone (IRS) is inadequate to ensure sufficient primary stability for an immediate molar implant (IMI), a viable alternative could involve situating the implant within the palatal root socket of the maxillary molar. This approach leverages the bone present at the apical region to achieve the necessary initial stability [4]. In a clinical investigation, 52 participants with a total of 61 maxillary molars were monitored over a 24-month period following immediate palatal root implant placement. The study reported a perfect implant survival rate of 100%. The mean marginal bone resorption observed at the 6-month mark was 0.12 ± 0.02 mm, with no instances surpassing 0.7 mm. By the 18-month check, the average marginal bone loss had increased to 0.19 ± 0.03 mm [6]. Furthermore, during the procedure of immediate molar implant placement in the palatal root socket of the maxilla, it is crucial to take into account the angle formed between the implant and the axis of occlusal force. Research indicates that employing a 20° inclined abutment in the posterior maxillary area is a theoretically viable option [5]. Nonetheless, given the variability in occlusal forces among individuals, there remains an inherent risk with the use of tilted implants. In this scenario, the angle of inclination was approximately 10°.

Immediate molar implant placement is performed without the foundation of soft tissue healing, making wound closure relatively difficult. The inability to suture the wound tightly initially may introduce some uncertainty to the primary healing of the wound, the stability of the bone substitute material, and the osseointegration of the implant. Some scholars have proposed that a personalized healing abutment can be used to assist in wound closure. The advantage of this method is that it can act as a mechanical barrier, stabilize the blood clot, and maintain the space required for bone regeneration. A randomized controlled clinical study randomly divided 36 patients into personalized and standardized healing abutment groups. The results at 4 months and 12 months postoperatively showed that the papilla index of the personalized group was significantly higher than that of the standardized group, and the margin bone loss in the standardized group was significantly higher than that in the personalized group [7]. Therefore, the use of personalized healing abutments is also beneficial for maintaining the papilla and bone levels of immediate implant placement. In this case, the flowable resin was used to directly fabricate the personalized healing abutment in the mouth, and other methods such as postoperative intraoral scanning and CAD/CAM chair-side printing can also be used to fabricate personalized healing abutments.

## Conclusions

Placing the implant into the fresh post-extraction palatal socket of an extracted maxillary molar offers a safe and minimally invasive alternative to traditional staged implant procedures. This method simplifies the treatment regimen by minimizing the requirement for repeated surgical procedures, which in turn shortens the total duration of treatment and reduces the related expenses.
